# Postprandial Metabolism, Inflammation, and Plasma Bile Acid Kinetics in a Rat Model: Implications for Translational Research

**DOI:** 10.1002/mnfr.70174

**Published:** 2025-07-22

**Authors:** Larissa Rodrigues, Carlos M. Donado‐Pestana, Tushar More, Mar Garcia‐Aloy, Gustavo M. Carneiro de Lima, Lucas X. Martins de Oliveira, Urska Vrhovsek, Karsten Hiller, Jarlei Fiamoncini

**Affiliations:** ^1^ Department of Food Science and Experimental Nutrition, School of Pharmaceutical Sciences University of São Paulo São Paulo Brazil; ^2^ Food Research Center, FoRC, School of Pharmaceutical Science University of São Paulo São Paulo Brazil; ^3^ Braunschweig Integrated Centre of Systems Biology University of Braunschweig Braunschweig Germany; ^4^ Metabolomics Unit, Research and Innovation Centre Fondazione Edmund Mach (FEM) San Michele all'Adige Italy

**Keywords:** bile acids, dietary challenge, inflammation, metabolomics, nutritional physiology, postprandial metabolism

## Abstract

The postprandial period is an opportunity window to assess metabolic phenotype, and its study is gaining popularity due to the wealth of information that can be uncovered when a dietary challenge is associated with the application of metabolomics approaches. Bile acids (BA) were recently identified as signaling molecules that display major changes in circulating levels following food intake. In this regard, a gap of information remains linking BA postprandial kinetics with their possible metabolic effects. This study aimed to characterizing a murine model for investigating postprandial metabolism and inflammation. Changes in plasma and hepatic markers of metabolism, inflammation and BA levels were assessed in male Sprague‐Dawley rats before and after the ingestion of an energy‐dense meal. Rats display postprandial alterations in circulating BA levels, with cholic acid constituting the predominant species (36%). These changes are accompanied by shifts in intermediates of energy metabolism and inflammatory markers, as demonstrated by a four‐fold increase in hepatic NF‐κB protein content, a key inflammatory transcription factor, two hours after food intake. Despite inherent species‐specific differences, this murine model represents a promising tool for studying postprandial modulation energy metabolism, establishing a pioneering framework for future investigations into the role of BA in postprandial metabolic responses.

## Graphical abstract

Sprague‐Dawley rats were used to investigate postprandial metabolic and inflammatory responses following the consumption of an energy‐dense meal. Time‐resolved plasma and liver analyses revealed fluctuations in bile acid levels, particularly cholic acid, along with changes in metabolic intermediates, oxylipins, and inflammatory markers. This study offers novel insights into diet‐induced physiological adaptations in a murine model and underscores the importance of understanding these responses for effectively translating preclinical findings.

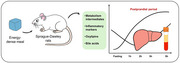

## 1 Introduction

The postprandial period involves physiological adjustments to accommodate the influx of energy substrates, influenced by meal composition and energy content. Typically lasting 2–3 h, it can extend up to 8 h for high‐fat meals [1].

During this time, absorbed nutrients, gut‐derived and endogenous metabolites, as well as endocrine signals, enter circulation, affecting cellular metabolism [2]. Dysregulated metabolic responses to food intake can lead to chronic diseases like diabetes and cardiovascular diseases [2, 3, 4]. Energy‐dense meals with rapidly absorbed nutrients can cause spikes in circulating glucose and triglycerides, a condition known as “postprandial dysmetabolism,” associated with endothelial dysfunction, oxidative stress, and hypercoagulation [1, 5]. A transient inflammatory response also occurs, linked to meal lipid content and endotoxin leakage from the intestine, marked by elevated inflammatory cytokines and leukocyte counts [6, 7, 8].

After the intake of a meal, circulating levels of bile acids (BA) increase 2–6‐fold up to 8 h, with significant interindividual variability [9]. BA‐specific receptors, widely expressed, position BA as postprandial signals influencing cellular functions, metabolism, and inflammation, with potential immunoregulatory effects [2, 10, 11]. Studies have explored BA's impact on metabolic diseases, including effects on body weight, insulin sensitivity, and glucose and lipid metabolism [12, 13, 14]. However, BA's influence on postprandial metabolic and inflammatory responses remains underexplored.

Studies investigating postprandial metabolism in humans predominantly focus on changes in plasma levels of metabolic intermediates and signaling molecules. However, these analyses seldom explore shifts in tissues such as the liver, intestine, and adipose tissue—key sites where energy metabolism is actively regulated. Reliance on plasma data alone limits insights into the processes activated or suppressed after food intake, as circulating metabolite levels cannot reveal their tissue‐specific origins or metabolic fates. Preclinical models, which allow direct tissue sampling, thus offer a critical advantage in unraveling the molecular mechanisms and organ‐level responses underlying postprandial adaptations.

In this report—the first from a study aimed at characterizing postprandial metabolic dynamics in rats—we compare food intake‐induced changes in plasma metabolites with those occurring in hepatic tissue. Additionally, given the emerging role of BA in metabolic regulation, we provide the first comprehensive analysis of postprandial kinetics for the 20 most abundant BA in plasma. By integrating tissue‐specific and systemic metabolic profiles, this work establishes a foundation for elucidating the contribution of BAs and organ‐level responses to postprandial physiology.

## 2 Material and Methods

### 2.1 Animals and Dietary Challenge

All experiments complied with Brazilian legislation and were approved by the Animal Ethics Committee of the University of São Paulo (FCF/USP/CEUA/629). Fifty male Sprague‐Dawley rats (11 weeks old) were housed under controlled conditions (21°C–23°C, 40%–60% humidity, 12 h light/dark cycle) with ad libitum access to a balanced chow diet and water. Rats were trained to eat a test meal spontaneously for 1 week. Spontaneous feeding replaces gavage and is an important strategy for this protocol, as it reduces animal stress and possible effects on metabolism [15]. The test meal included gelatin cubes (4 mL) containing carbohydrates (sucrose), lipids (soybean oil), protein (casein), agar (1.2%), bacon flavor, and an edible blue dye. The meal provided ∼43 kcal, equivalent to ∼50% of an adult rat's daily energy requirement [16], with ∼60% of energy from lipids.

The rats were trained to ingest the test meal by being exposed to it daily during the week prior to the experiment. The day before the dietary challenge, the animals were fasted overnight. On the test day, the meal was presented at 8:00 a.m., and all animals consumed the entire portion within a 15‐min period. Groups of 10 rats were euthanized at 0 (fasting), 60, 120, 180, and 300 min postfeeding. Blood samples (via cardiac puncture), liver, and other tissues were collected at these time points.

### 2.2 Plasma Markers of Intermediate Metabolism

Plasma was separated from blood collected in EDTA‐treated tubes and analyzed for total cholesterol, triacylglycerols, glucose, and nonesterified fatty acids (NEFA) using commercial kits (LABTEST and FUJIFILM Wako Chemicals). Insulin, peptide YY (PYY), and gastric inhibitory polypeptide (GIP) levels were measured using immunoassay analysis (Milliplex, Merck).

### 2.3 Protein Analysis by Immunoblotting

Liver samples (∼100 mg) were homogenized in lysis buffer, centrifuged, and protein concentration was determined using the Pierce BCA Protein Assay Kit (Thermo Scientific, Rockford, USA). Proteins were separated by SDS‐PAGE, transferred to PVDF membranes, and incubated with primary antibodies for TLR‐4, NF‐κB, SAPK/JNK, IKKβ, IL‐6, and β‐actin (Cell Signaling Technology, Beverly, USA). Membranes were incubated with peroxidase‐conjugated secondary antibodies and developed using enhanced chemiluminescence (ECL) substrate (Merck Millipore, Massachusetts, USA). Band densities were quantified using ImageJ software (National Institute of Health, USA).

### 2.4 Gene Expression Analysis by Quantitative PCR

mRNA from liver samples was extracted using Trizol (Life Technologies, Thermo Scientific, Waltham, MA, USA) and purified with the PureLink RNA Mini kit (Invitrogen, Thermo Fisher Scientific, USA). RNA was quantified on a NanoDrop spectrophotometer (Thermo Scientific, Waltham, MA, USA) and reverse transcribed to cDNA with a High‐Capacity Reverse Transcription kit (Applied Biosystems, Thermo Fisher Scientific, USA). Real‐time PCR was performed using SYBR Green JumpStart Taq ReadyMix (Sigma Aldrich, St. Louis, MO, USA) on a StepOnePlus Real‐Time PCR System (Applied Biosystems, Thermo Fisher Scientific, USA). Four reference genes were tested as housekeeping: the ribosomal genes HPRT1, RPLP0, and 18S, and the nuclear gene B2M (Table 1). Hypoxanthine phosphoribosyltransferase 1 (HPRT1) was used as internal standard due to its lowest variation and high degree of reproducibility, homogeneity, and expression stability among the different time points. Gene expression was quantified using the ΔΔCt method.

### 2.5 Enzyme‐Linked Immunosorbent Assay (ELISA)

Liver homogenates (∼100 mg) were prepared as for immunoblotting, and IL‐6 and TNF‐α levels were measured using ELISA kits (DuoSet ELISATM, R&D Systems, Minneapolis, MN, USA).

### 2.6 Gas Chromatography‐Mass Spectrometry (GC‐MS)‐Based Metabolite Profiling

Metabolite extraction and GC‐MS analysis were performed according to More et al. [17]. Metabolites were extracted from plasma (10 µL) or liver (10 mg) using methanol (8:1) with internal standards U13C‐ribitol and D6‐glutaric acid. The extract was dried in a centrifugal vacuum concentrator and derivatized in two steps. GC‐MS analysis was conducted on an Agilent 7890A GC with a DB‐35MS column, helium as the carrier gas, and full scan mass spectra were acquired. QC samples were used for data correction [18], and compound identification was based on an in‐house mass spectral library [19].

### 2.7 Untargeted Metabolomics Analysis Using LC‐HRMS

Plasma (50 µL) was processed using an Ostro 96‐well plate and methanol, then filtered with acetonitrile + 1% formic acid. The dried extract was re‐dissolved in acetonitrile (1:1 V/V) and analyzed on a Dionex UltiMate 3000 HPLC system coupled to a hybrid linear ion trap Fourier transform Orbitrap mass spectrometer (Thermo Fisher, Bremen, Germany), following the method previously described by Garcia‐Aloy et al. [20]. QC and blank samples were interspersed throughout the analysis for quality control. Data files were processed using XCMS and Spectra packages in R, and metabolites were identified with in‐house and online mass spectral libraries. Metabolites were filtered based on signal quality, variability, and response to dilutions, retaining those with adjusted *R* ^2^ > 0.5 on a 7‐point QC dilution curve.

### 2.8 Bile Acid Analysis

Deuterated bile acid standards were used to quantify plasma concentrations of bile acids. The experimental procedure was an adaptation of a previously described method and involved the use of a liquid chromatography‐mass spectrometry (LC‐MS) technique for the separation and quantitation of bile acids. A volume of 10 µL of plasma was deproteinized with methanol containing labeled standards, centrifuged, and dried. The extract was reconstituted in methanol (1:1) and analyzed on a UPLC system equipped with a Waters Acquity UPLC HSS T3 column. Separation and quantification were achieved using a triple quadrupole mass spectrometer (5500 Sciex, MA, USA) in negative mode with multiple reaction monitoring. Data analysis was performed using Analyst software (Sciex).

**TABLE 1:** List of primer sequences used.

Gene	Forward sequence	Reverse sequence
*NF‐κB*	TTCAACATGGCAGACGACGA	TGGGGGCTTTGCTGTCATAG
*IL‐6*	ACAAGTCCGGAGAGGAGACT	GAATTGCCATTGACAAACTCT
*IL‐1β*	CAGCTTTCGACAGTGAGGAGA	TGTCGAGATGCTGCTGTGAG
*IFN‐γ*	TGTCATCGAATCGCACCTGA	TGTGGGTTGTTCACCTCGAA
*TGF‐β*	CTGCTGACCCCCACTGATA	AGCCCTGTATTCCGTCTCCT
*HPRT1*	CAGTCCCAGCGTCGTGATTAG	GCACACAGAGGGCCACAATG
*RPLP0*	TCGAAGCAAAGGAAGAGTCGG	TTAAGCAGGCTGACTTGGTGTG
*B2M*	TTCCACCCACCTCAGATAGAAAT	TGTGAGCCAGGATGTAGAAAGAC
18S	GGGAGGTAGTGACGAAAAATAACAAT	TTGCCCTCCAATGGATCCT

Abbreviations: *18S*, 18S ribosomal RNA; *B2M*, β2 microglobulin; *HPRT1*, hypoxanthine phosphoribosyltransferase 1; *IL‐1β*, interleukin 1 beta; *IL‐6* interleukin 6, *IFN‐γ*, interferon gamma; *NF‐κB*, necrosis, nuclear factor‐kappa B; *RPLP0*, ribosomal protein lateral stalk subunit P0; *TGF‐β*, transforming growth factor‐β.

### 2.9 Statistical Analysis

Results were expressed as mean ± standard error (SEM). Data were assessed for normality using the Shapiro–Wilk test. Statistical differences among groups were evaluated using one‐way ANOVA with Tukey's post hoc test for normally distributed data, or Kruskal–Wallis with Dunn's post hoc test for nonparametric data. For metabolomics data, differences among groups were evaluated using one‐way ANOVA followed by False Discovery Rate (FDR) correction to account for multiple testing. Specifically, the two‐stage linear step‐up procedure of Benjamini, Krieger, and Yakutieli, as implemented in GraphPad Prism software (version 9.0, La Jolla, CA, USA), was applied. This adaptive method assumes that the test statistics are independent or positively correlated and improves statistical power by first estimating the proportion of true null hypotheses. Metabolites with an adjusted *p* value (*q* value) less than 0.05 were selected (except for glycerol, which was included due to its biological relevance despite marginal significance). As each postprandial timepoint was assessed using a distinct set of animals, one‐way ANOVA was appropriate rather than repeated‐measures ANOVA. Table S1 reports the adjusted *p* values and *q* values for all pairwise comparisons of metabolomic data, providing a comprehensive overview of the statistical results.

## 3 Results

### 3.1 Gastrointestinal Transit and Postprandial Plasma Biochemical Profile

**FIGURE 1:**
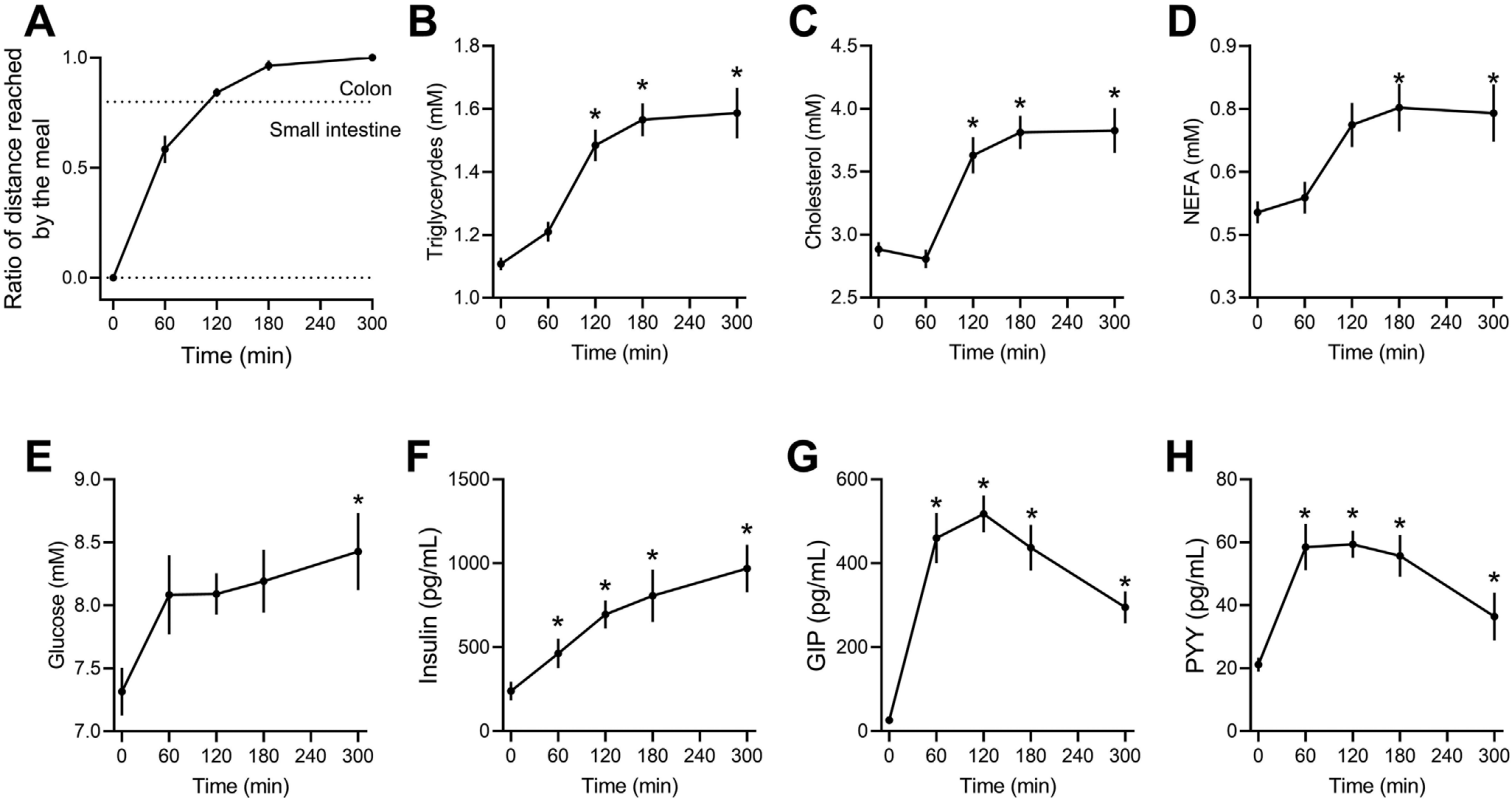
Gastrointestinal transit time and circulating markers of intermediate metabolism. (A) Distance reached by the meal in the hours following the ingestion of the energy‐dense meal. (B) Plasma triglycerides. (C) Total cholesterol. (D) Nonesterified fatty acids. (E) Glucose. (F) Insulin. (G) Gastric inhibitory polypeptide (GIP). (H) Peptide YY (PYY). Data were analyzed by one‐way ANOVA followed by Tukey's multiple comparison test and expressed as mean ± SEM (*n* = 8 from biological replicates). ^*^ *p* < 0.05 in comparison to fasting.

Using the length of the gastrointestinal (GI) tract stained by a blue dye in the meal, the average GI transit time in rats was observed to be between 3 and 4 h (Figure 1A and Figure S1). With a similar kinetic profile, plasma triglycerides, total cholesterol, and NEFA significantly increased 2 h after the test meal and remained elevated up to the fifth hour (*p* < 0.0001, *p* = 0.0005, and *p* = 0.0313, respectively; Figures 1B–D). Plasma glucose levels remained relatively stable postprandially, increasing by ∼15% despite an intake of approximately 4 g glucose/kg body weight (*p* = 0.0133, Figure 1E). Insulin levels rose significantly in the first hour postmeal, reaching a fivefold increase by the fifth hour compared to the fasting state (*p* < 0.0001, Figure 1F). Plasma levels of GIP and PYY increased after meal intake, peaking at 60 min, with GIP showing a 50‐fold increase (*p* < 0.0001) and PYY a threefold increase (*p* = 0.0004) compared to fasting. Notably, GIP levels remained 30 times higher than fasting levels even 5 h postmeal (Figures 1G,H).

### 3.2 Postprandial Alterations of Plasma and Hepatic Metabolome

**FIGURE 2:**
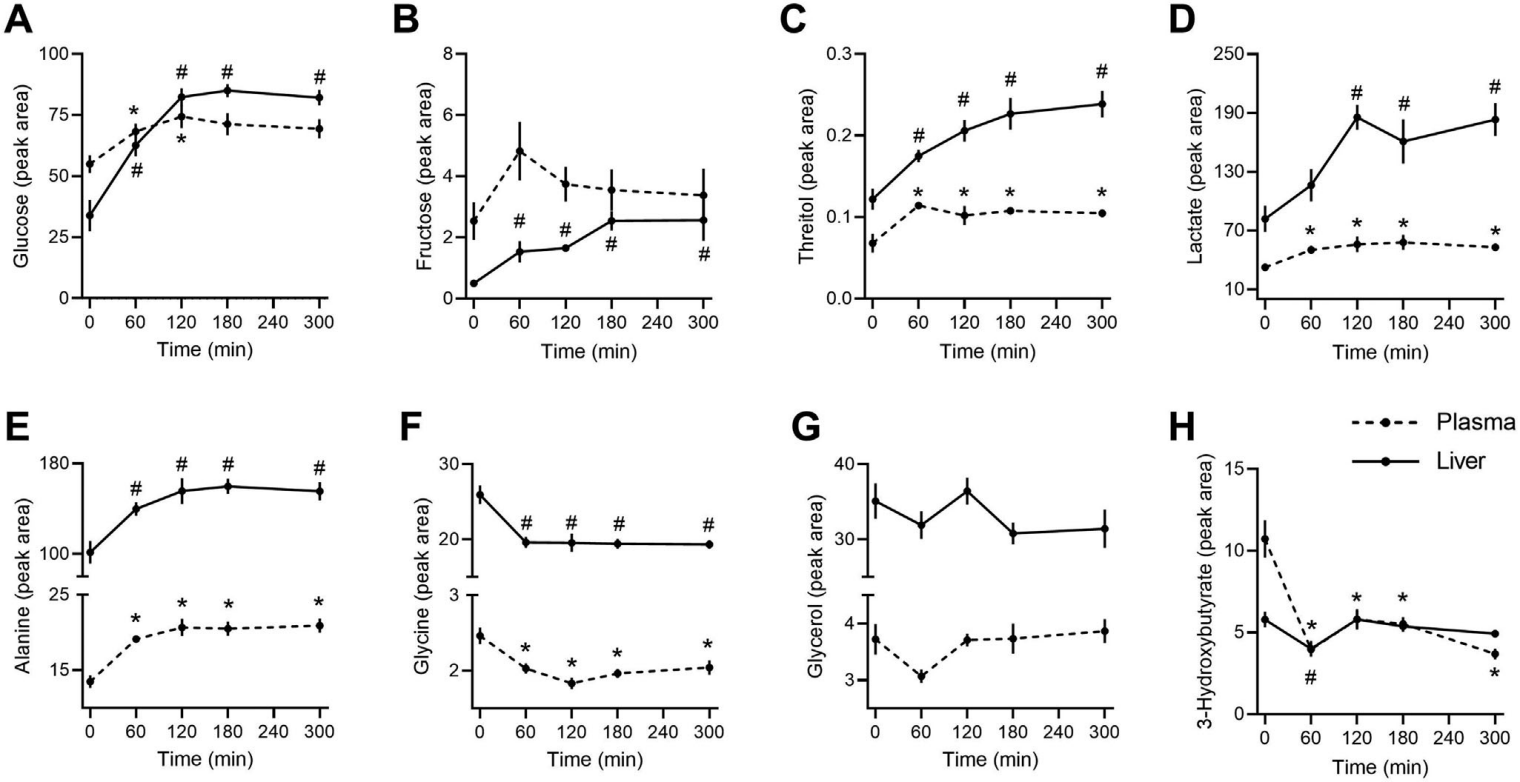
Plasma and liver metabolites identified through GC/MS. (A) glucose, (B) fructose, (C) threitol, (D) lactate, (E) alanine, (F) glycine, (G) glycerol, and (H) 3‐hydroxybutyrate. Data were analyzed by one‐way ANOVA, adjusted by FDR, and expressed as mean ± SEM of normalized peak areas (*n* = 8–10 from biological replicates). ^*^ *p* < 0.05 compared to fasting for plasma markers, ^#^ *p* < 0.05 compared to fasting for liver markers.

Untargeted metabolomics using GC‐MS identified 191 metabolites in plasma and 98 in the liver, with 58% identified overall (68% of plasma and 47% of liver metabolites). The postprandial profile of identified metabolites was similar in both plasma and liver. Hepatic glucose reached its maximum at 120 min postmeal, showing a 140% increase compared to fasting (*p* < 0.0001, Figure 2A), while plasma glucose increased modestly (∼15%). Fructose levels slightly rose in plasma but showed a fourfold increase in the liver (Figure 2B). Threitol, a product of xylose metabolism, increased in both plasma and liver during the postprandial period (Figure 2C). Hepatic lactate levels more than doubled, reaching their peak at 120 min (Figure 2D). Gluconeogenic amino acids such as alanine increased by 60%, while glycine decreased by 50% in both plasma and liver (Figures 2E,F). Consistent with reduced ketogenesis postprandially, 3‐hydroxybutyric acid (3HBA) levels decreased by 25% in plasma and 70% in the liver after the test meal (Figures 2G,H).

**FIGURE 3:**
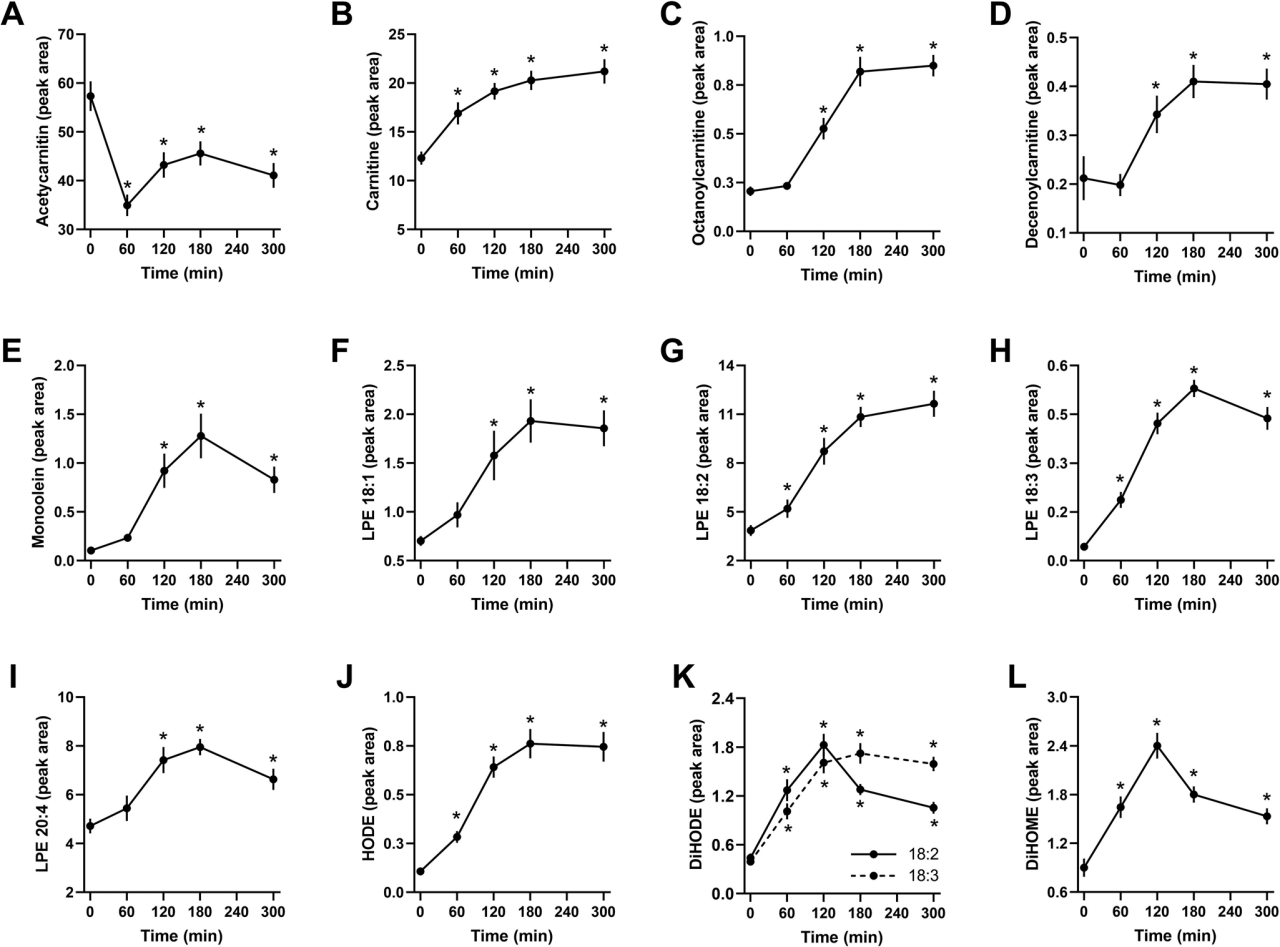
Plasma metabolites identified using UPLC/HRMS. (A) Acetylcarnitine, (B) carnitine, (C) octanoylcarnitine, (D) decenoylcarnitine, (E) monoolein, (F) LPE 18:1, (G) LPE 18:2, (H) LPE 18:3, (I) LPE 20:4, (J) HODE, (K) DiHODE 18:2 and 18:3, and (L) DiHOME. Data were analyzed by ANOVA, adjusted by FDR, and expressed as mean ± SEM of peak areas × 10^6^ (*n* = 8–10 from biological replicates). ^*^ *p* < 0.05 compared to fasting.

Further analysis using high‐resolution LC‐MS identified 91 plasma metabolites, including lysophosphatidylethanolamines (LPE), oxylipins, and acylcarnitines. Plasma acetylcarnitine levels decreased 1 h postmeal (*p* < 0.0001, Figure 3A). Free carnitine and medium‐chain acylcarnitines (C8 and C10) followed a similar pattern to NEFA and triglycerides, increasing from 120 min onward (*p* = 0.0012, *p* = 0.0002, and *p* = 0.0394, respectively, Figures 3B–D). Plasma levels of monoolein, a monoacylglycerol from triglyceride digestion, increased 13‐fold at 180 min (*p* < 0.0001, Figure 3E). LPEs (18:1, 18:2, 18:3, and 20:4) showed significant increases from the second hour postmeal, remaining elevated until the fifth hour (Figures 3F–I). Oxylipins such as HODE, DiHODE (18:2 and 18:3), and DiHOME rose several‐fold, peaking at the second hour of the dietary challenge (Figures 3J–L).

### 3.3 Postprandial Hepatic Inflammation

**FIGURE 4:**
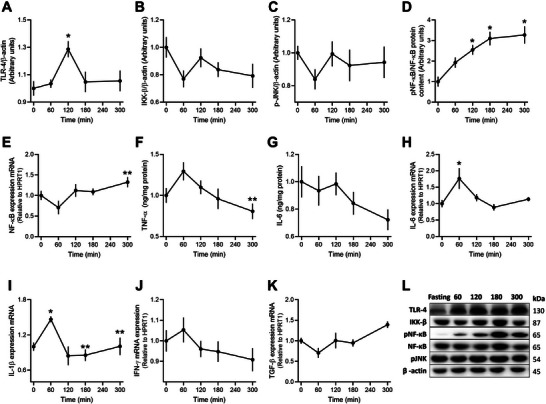
Markers of inflammation in the liver. (A) TLR‐4 protein content, (B) IKKβ protein content, (C) JNK protein content, (D) protein content of NF‐κB, (E) mRNA expression of NF‐κB, (F) TNF‐α level, (G) IL‐6 content, (H) mRNA expression of IL‐6, (I) mRNA expression of IL‐1β, (J) IFN‐γ mRNA expression, (K) TGF‐β mRNA expression, and (L) representative immunoblots. Data were analyzed by one‐way ANOVA followed by Tukey's multiple comparison test and expressed as mean ± SEM (*n* = 8 from biological replicates). ^*^ *p* < 0.05 compared to fasting, ^**^ *p* < 0.05 compared to 60 min. HPRT1 was used as housekeeping in the mRNA expression analysis.

Hepatic Toll‐like receptor 4 (TLR4) increased by 30% 2 h postmeal (*p* = 0.0206, Figure 4A). Inhibitor of nuclear factor kappa‐B kinase subunit beta (IKK‐B) and c‐Jun N‐terminal kinases (JNK) did not show significant changes, although there was a trend for reduced content 1 h postmeal (Figures 4B,C). The active phosphorylated form of NF‐κB increased fourfold 2 h postmeal and continued to rise until the fifth hour (*p* < 0.0001, Figure 4D). NF‐κB mRNA expression was elevated at 300 min compared to 60 min (*p* = 0.018, Figure 4E). Hepatic levels of TNF‐α and IL‐6 decreased slightly by the fifth hour compared to the fasted state (Figures 4F,G). IL‐6 and IL‐1β gene expression increased at 60 min (*p* = 0.0222 and *p* = 0.0166, respectively, Figures 4H,I). IFN‐γ and TGF‐β gene expression remained unchanged postmeal (Figures 4J,K).

### 3.4 Postprandial Kinetics of Bile Acids in Plasma

**FIGURE 5:**
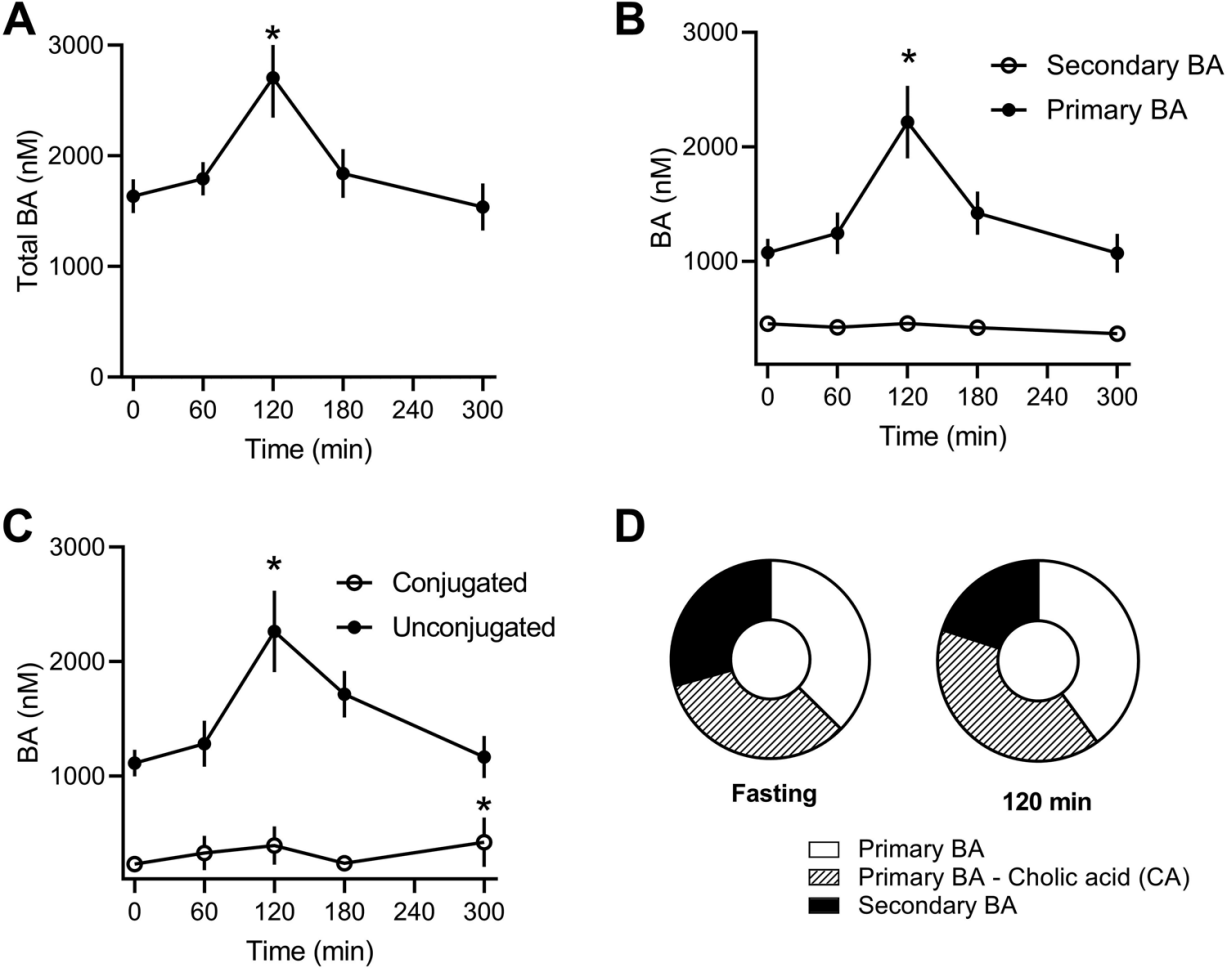
Plasma bile acid profile. (A) Total BA, (B) primary and secondary BA, (C) conjugated and unconjugated BA, (D) percentual composition of plasma bile acids at fasting and 120 min after food intake highlighting the participation of cholic acid. Data were analyzed by ANOVA followed by Tukey's multiple comparison test and expressed as mean ± SEM (*n* = 8 from biological replicates). ^*^ *p* < 0.05 compared to fasting.

Twenty BA species were quantified in plasma, with total BA increasing by 65% at 120 min postmeal (*p* = 0.0093, Figure 5A). Primary BAs, especially unconjugated ones, were the most abundant and increased the most postmeal, while secondary BAs showed no significant postprandial changes (Figure 5B). Unconjugated BAs doubled in concentration 2 h postmeal (*p* = 0.0022), whereas conjugated BAs remained largely unchanged (Figure 5C). Primary‐unconjugated BAs predominated, with cholic acid being the most prevalent, accounting for approximately 40% of total BAs (Figure 5D and Figure S2).

## 4 Discussion

Understanding how meal ingestion affects metabolic responses during the postprandial period is particularly important, as it provides valuable insights into how dietary components and patterns influence the risk of developing chronic diseases. The effects of such responses remain largely unexplored at specific tissue sites. Instead, in the vast majority of studies involving human and animal models, postprandial homeostasis is captured via time‐resolved blood sampling. We have established a meal‐fed rat model designed to facilitate the characterization of biochemical and metabolite signatures during postprandial homeostasis in plasma and the liver, as the liver plays a central role in energy regulation following nutrient intake. Our study provides novel insights, clearly demonstrating for the first time that Sprague‐Dawley rats exhibit postprandial responses similar to those observed in humans. This murine model reveals a transient inflammatory response, evidenced by increased inflammatory markers in both circulation and the liver. Despite species‐specific differences that must be considered, rats also display changes in circulating bile acid (BA) levels during the postprandial phase, with a predominance of cholic acid.

Our findings on gastrointestinal (GI) transit time align with previous reports, indicating that 3–4 h are required for ingested food to reach the large intestine and be converted into fecal pellets in rats, which is considerably shorter than the average 26 h observed in humans [21, 22]. Despite this difference in transit time, the postprandial plasma kinetics of triglycerides in the murine model closely resemble those observed in humans challenged with a high‐fat meal, which show a 30%–50% increase in plasma triglyceride levels [23, 24]. Unlike the unaltered postprandial levels of cholesterol observed in humans [25], rats displayed increased cholesterol levels during the absorptive phase. This response may be linked to the lack of cholesteryl ester transfer protein (CETP) activity in rodents, a key enzyme in human cholesterol transport [26], and warrants further investigation. The increased postprandial plasma levels of NEFA observed in rats are distinct from the oscillations seen in humans, where NEFA levels typically decrease within the first couple of hours after a meal due to insulin‐mediated inhibition of hormone‐sensitive lipase (HSL) in adipose tissue, with levels rebounding later as lipoprotein lipase (LPL) activity increases [27, 28]. This unexpected result in rats requires further exploration.

Rats also showed a prolonged increase in insulin levels until the fifth hour postmeal, in contrast to humans, where insulin typically spikes within the first hour after a mixed meal and declines thereafter [29]. Insulin plays a crucial role in regulating metabolic responses to nutrient consumption and is considered the master regulator of postprandial metabolic adaptations [30, 31]. Despite the distinct insulinemic response, the excursions of GIP and PYY in rats were very similar to humans, with GIP showing a more pronounced increase than PYY [32, 33].

Interpreting metabolomics data, which reflect concentrations of metabolic intermediates influenced by both their production and clearance rates, is inherently complex. In this study, we compared the postprandial profiles of metabolites in plasma and liver. Since the liver is the first organ to receive newly absorbed polar nutrients, postprandial increases in glucose, fructose, pyruvate, and alanine were more pronounced in the liver than in plasma. For instance, hepatic glucose levels rose by 140% at 120 min and remained elevated until the fifth hour. Similar patterns were observed for fructose and threitol, a product of xylose metabolism, which have been linked to increased plasma levels in diabetic individuals and high‐fat diet‐fed rats [34, 35]. Lactate and alanine, which also showed postprandial increases, reflect a similar profile in both rats and humans [36, 37]. However, plasma levels of glycine, leucine, isoleucine, and threonine decreased (Figure S1), likely driven by increased protein synthesis during the postprandial period, which is upregulated by insulin signaling [38, 39, 40, 41].

As expected, ketogenesis was suppressed in the postprandial period, as shown by decreased plasma and hepatic levels of 3‐hydroxybutyrate (3HBA). In humans, 3HBA levels typically drop shortly after a high‐fat meal and then rebound, reaching levels much higher than the fasting state [42, 43]. This rebound was not observed in the plasma of rats, although hepatic 3HBA levels did show a modest increase after the first hour, possibly due to sustained insulin elevation suppressing fatty acid oxidation.

Acylcarnitines, which mirror acyl‐CoA levels from amino acid degradation or fatty acid β‐oxidation, showed a postprandial decrease in acetylcarnitine levels, similar to humans [44, 45, 46]. However, at 2 h postmeal, plasma levels of free carnitine and medium‐chain acylcarnitines like octanoylcarnitine (C8) and decanoylcarnitine (C10) increased in rats, whereas in humans these levels generally decrease postprandially [47]. This discrepancy suggests that β‐oxidation of fatty acids in rats may not be inhibited to the same extent as in humans [48], and the mechanisms underlying these differences merit further study.

Phospholipids, including lysophosphatidylcholine (PC) and lysophosphatidylethanolamines (LPE), are crucial for cellular structure, function, and lipoprotein assembly [49]. Plasma lysophospholipids, likely derived from lecithin‐cholesterol acyltransferase (LCAT) and secretory phospholipases, showed postprandial increases consistent with findings in humans. Changes in these lipids have been linked to metabolic alterations in obesity, suggesting potential implications for lipid metabolism disorders [50, 51].

Oxylipins are involved in many biological processes and participate in the regulation of apoptosis, cell proliferation, inflammation, immune actions, tissue repair, blood vessel permeability, and blood pressure regulation [52]. In our study, the postprandial period in rats was marked by an increase in oxylipin levels. This phenomenon may be partly attributed to the consumption of the lipid component of the meal (soybean oil), which is primarily composed of linoleic acid, a major precursor in the synthesis of oxylipins [53]. Similarly to our observations, a previous study found a remarkable increase in the abundance of oxylipins in response to the ingestion of a high‐fat meal. The authors identified a postprandial oxylipin signature circulating in triglyceride‐rich lipoproteins in pro‐atherogenic/dyslipidemic subjects [54, 55].

Postprandial inflammatory markers, such as cytokines, are transient and highly variable, making them difficult to capture in systemic circulation [56, 57]. To address this, we examined hepatic inflammation, finding time‐resolved increases in TLR4 protein content and NF‐κB mRNA expression. TLR4, which is activated by lipopolysaccharides and saturated fatty acids, plays a key role in initiating inflammation in response to dietary fat intake [58, 59, 60]. The increase in NF‐κB phosphorylation and subsequent gene expression of pro‐inflammatory cytokines like IL‐6 and IL‐1β at 60 min postmeal suggests a rapid inflammatory response in the liver, potentially driven by absorbed nutrients and circulating NEFA levels.

In humans, BAs are among the metabolites with the greatest postprandial variation [61]. Following an oral glucose tolerance test (OGTT) or a high‐fat meal, plasma BA levels rise rapidly, predominantly as glycine‐conjugated species [9]. In contrast, rats show a predominance of primary, unconjugated BAs, with cholic acid representing a significant proportion. While humans mainly conjugate BAs with glycine [62], rats favor taurine‐conjugation, a difference noted in both rats and mice [63, 64]. Despite the lack of a gallbladder [65], rats exhibited a postprandial increase in total plasma BA levels, primarily driven by unconjugated primary BAs such as cholic and chenodeoxycholic acids. Cholic acid was the main BA species found in the plasma of rats, both in the fasting state and postprandially. It demonstrated a significant increase after 120 min (Figure S2A), being the major contributor to the overall increase in plasma BA after food intake. Previous studies describing the plasma profile of BA in rodents also showed cholic acid as the most abundant BA [63]. In Sprague‐Dawley rats, plasma levels of BA conjugated to glycine or taurine remained largely unchanged during the postprandial period. Despite differences in GI transit, BA *T*_max_ is the same in rats and humans at 120 min. In rats, total BA levels in plasma return to initial values at the fifth hour, while humans tend to sustain higher levels of BA for periods longer than 8 h after ingesting a high‐fat, high‐sugar meal [62, 66].

This comprehensive examination of postprandial BA kinetics in Sprague‐Dawley rats highlights significant differences in the metabolic response to food intake between humans and rats. Despite the consumption of a high‐fat meal, the increase in circulating BA levels in rats was modest, transient, and specific to certain BA classes. In contrast, humans typically exhibit several‐fold changes in plasma BA levels under similar conditions [62]. We hypothesize that the absence of a gallbladder in rats might partly explain this effect. A similar pattern is observed in humans who have undergone surgical gallbladder removal, which exhibit a slight postprandial increase in plasma BA compared to control subjects [67]. Variations in postprandial kinetics, conjugation patterns, and predominant BA species underscore the need to consider these differences when translating findings from animal models to humans. Overall, our study provides valuable data on the metabolic and inflammatory responses in rats, offering a basis for further research into the complex interactions governing postprandial physiology in both rodents and humans.

In this study, we describe postprandial metabolic and inflammatory responses in Sprague‐Dawley rats. While the model successfully presents evidence of postprandial inflammation and exhibits relatively similar kinetics for most of the metabolites measured as compared to humans, important species‐specific differences were observed, such as for postprandial changes in plasma insulin and cholesterol levels. Anatomical differences between humans and rats, such as the absence of a gallbladder and the rapid gastrointestinal transit time in rats, are also factors that must be considered when using rats to investigate postprandial physiology. Additionally, further studies are required to expand our findings by including analysis of other key metabolic organs, which would enable a comprehensive understanding of tissue‐specific postprandial response. Despite these limitations, the current model provides valuable insights that contribute to the understanding of postprandial metabolism.

## 5 Conclusion

This study provides novel insights and data derived from the characterization of a rat model that supports its applicability for the investigation of postprandial inflammation and metabolic changes. Sprague‐Dawley rats display metabolic responses during the postprandial period that are similar to what is observed in humans, mainly increased plasma levels of triglycerides, glucose, incretins, and BA, as well as responses that are clearly different in relationship to humans, which include the time‐resolved profiles of NEFA, cholesterol, and insulin. Sprague‐Dawley rats display postprandial inflammation, revealed by elevated pro‐inflammatory cytokines and inflammatory markers in the liver and in plasma. These species‐specific characteristics in postprandial metabolism must be carefully considered when interpreting preclinical data and translating findings from rodent models to humans. Despite the reported differences, we believe that Sprague‐Dawley rats can serve as a model to investigate the relationship between postprandial increase in BA levels and transient changes in energy metabolism and inflammation.

## Conflicts of Interest

The authors declare no conflicts of interest.

## Supporting information




**Supporting File 1**: mnfr70174‐sup‐0001‐SuppMat.docx.

## Data Availability

The data that support the findings of this study are available from the corresponding author upon request.
