# Supplementary material for: Postprandial Metabolism, Inflammation, and Plasma Bile Acid Kinetics in a Rat Model: Implications for Translational Research
Source: Mol Nutr Food Res. 2025 Jul 22;69(20):e70174. doi: 10.1002/mnfr.70174 (PMC12538531; doi:10.1002/mnfr.70174)
Supplement: Supplementary file 1 — Supporting File 1: mnfr70174‐sup‐0001‐SuppMat.docx. [file MNFR-69-e70174-s001.docx]

**
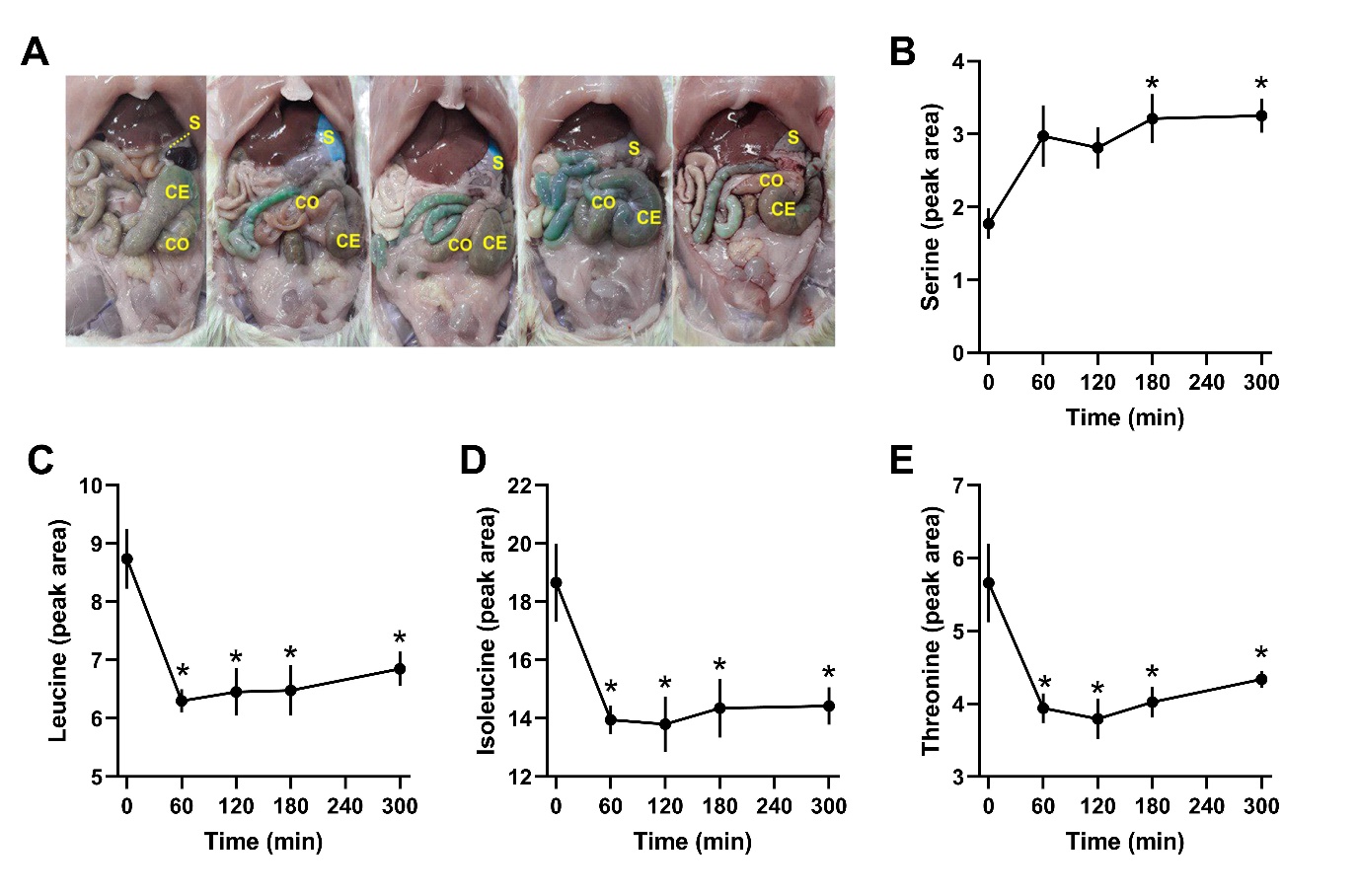
**

**Supplementary figure 1**. Postprandial progression of the meal though the gastrointestinal tract and hepatic amino acids analyzed by GC/MS. (A) Abdominal organs from rats euthanized at sequential intervals following the ingestion of a meal-colored blue (the images depict the stomach (S), cecum (CE), and colon (CO)), (B) Serine, (C) Leucine, (D) Isoleucine, (E), Threonine. Data were analyzed by one-way ANOVA followed by Tukey’s multiple comparison test and expressed as mean ± SEM (n = 6-10 from biological replicates). * p < 0.05 compared to fasting (plasma), # = p < 0.05 compared to fasting (liver).

**
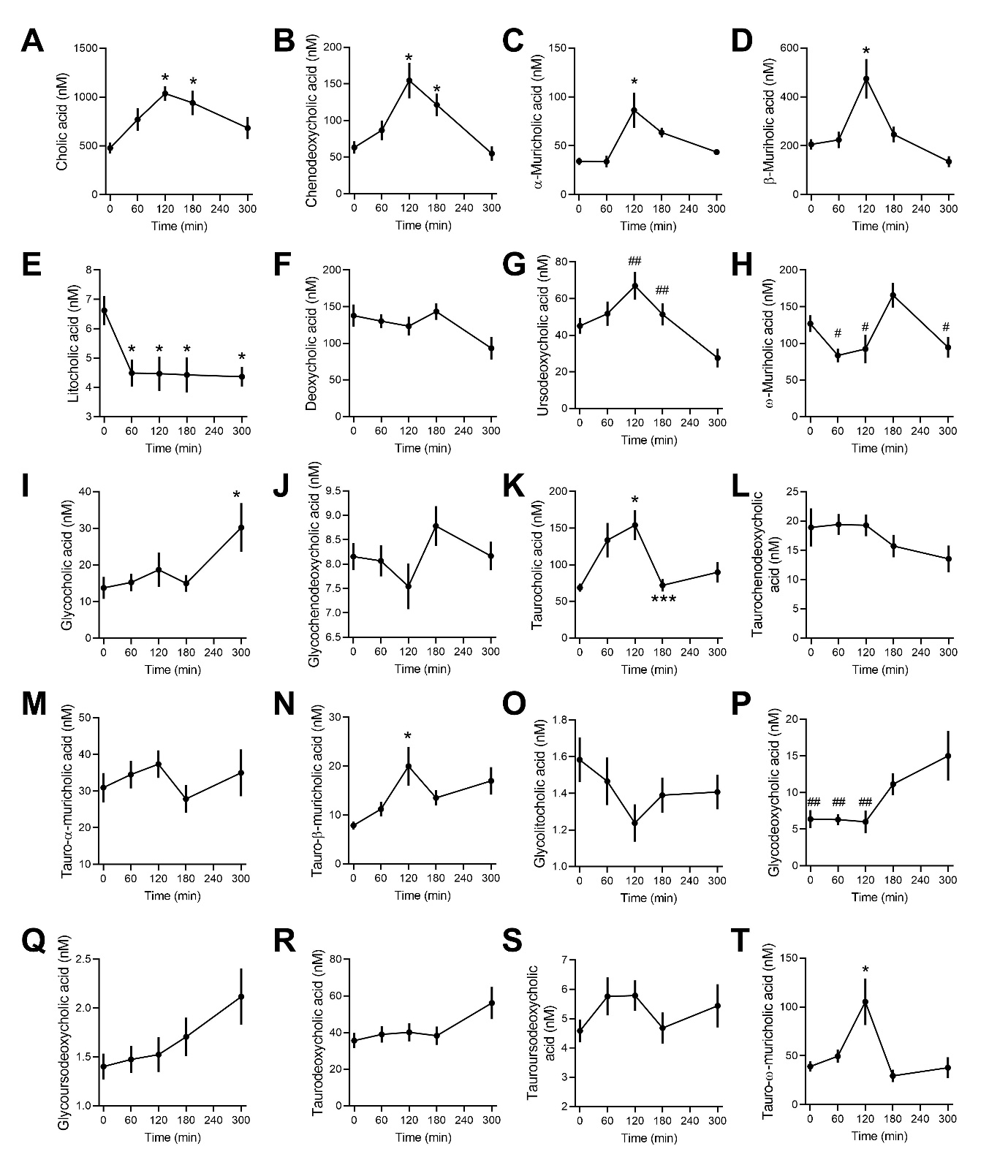
**

**Supplementary figure 2**. Plasma levels of individual bile acids during the dietary challenge. (A) Cholic acid, (B) Chenodeoxycholic acid (C) α-Muricholic acid, (D) β-Muricholic acid, (E) Lithocholic acid, (F) Deoxycholic acid, (G) Ursodeoxycholic acid, (H) ω-Muricholic acid, (I) Glycocholic acid, (J) Glycochenodeoxycholic acid, (K) Taurocholic, (L) Taurochenodeoxycholic acid, (M) Tauro-α-Muricholic acid (N) Tauro- β-Muricholic acid, (O) Glycolithocholic acid,(P) Glycodeoxycholic acid,(Q) Glycoursodeoxycholic acid, (R) Taurodeoxycholic acid, (S) Tauroursodeoxycholic acid, (T) Tauro- ω-Muricholic acid. Data were analyzed by one-way ANOVA followed by Tukey’s multiple comparison test and expressed as mean ± SEM (n = 8 from biological replicates). * *p* < 0.05 compared to fasting, ** *p* < 0.05 compared to 60 min, *** *p* < 0.05 compared to 120 min, # *p* < 0.05 compared to 180 min, ## *p* < 0.05 compared to 300 min.

**Supplementary table 1.** Adjusted *p*- and *q*-values for multiple comparisons of identified metabolites in postprandial period.

| **Figure** | **Metabolite** | **Multiple comparisons** | | | | | | | |
| --- | --- | --- | --- | --- | --- | --- | --- | --- | --- |
|  |  | Fasting vs. 60 min | | Fasting vs.120 min | | Fasting vs.180 min | | Fasting vs.300 min | |
|  |  | ***q*** | ***p*** | ***q*** | ***p*** | ***q*** | ***p*** | ***q*** | ***p*** |
| 2.A | Glucose - Plasma | 0.0318 | 0.0303 | 0.0110 | 0.0026 | 0.0185 | 0.0088 | 0.0270 | 0.0193 |
|  | Glucose - Liver | <0.0001 | <0.0001 | <0.0001 | <0.0001 | <0.0001 | <0.0001 | <0.0001 | <0.0001 |
| 2.B | Fructose - Plasma | 0.2079 | 0.0495 | 0.4834 | 0.3034 | 0.4834 | 0.3742 | 0.4834 | 0.4604 |
|  | Fructose - Liver | 0.0380 | 0.0725 | 0.0380 | 0.0592 | 0.0014 | 0.0013 | 0.0014 | 0.0007 |
| 2.C | Threitol - Plasma | 0.0009 | 0.0002 | 0.0051 | 0.0048 | 0.0025 | 0.0012 | 0.0035 | 0.0025 |
|  | Threitol - Liver | 0.0119 | 0.0113 | 0.0003 | 0.0002 | <0.0001 | <0.0001 | <0.0001 | <0.0001 |
| 2.D | Lactate - Plasma | 0.0394 | 0.0375 | 0.0208 | 0.0099 | 0.0183 | 0.0044 | 0.0277 | 0.0198 |
|  | Lactate - Liver | 0.0398 | 0.1516 | <0.0001 | 0.0002 | 0.0010 | 0.0028 | <0.0001 | 0.0001 |
| 2.E | Alanine - Plasma | <0.0001 | <0.0001 | <0.0001 | <0.0001 | <0.0001 | <0.0001 | <0.0001 | <0.0001 |
|  | Alanine - Liver | 0.0026 | 0.0024 | 0.0002 | 0.0001 | <0.0001 | <0.0001 | <0.0001 | <0.0001 |
| 2.F | Glycine - Plasma | 0.0011 | 0.0008 | <0.0001 | <0.0001 | 0.0003 | 0.0002 | 0.0012 | 0.0011 |
|  | Glycine - Liver | <0.0001 | <0.0001 | <0.0001 | <0.0001 | <0.0001 | <0.0001 | <0.0001 | <0.0001 |
| 2.G | Glycerol - Plasma | 0.2057 | 0.0490 | >0.9999 | 0.9589 | >0.9999 | 0.9736 | >0.9999 | 0.6343 |
|  | Glycerol - Liver | 0.3818 | 0.2727 | 0.7071 | 0.6734 | 0.3818 | 0.1958 | 0.3818 | 0.2181 |
| 2.H | 3-hydroxybutyrate - Plasma | <0.0001 | <0.0001 | <0.0001 | <0.0001 | <0.0001 | <0.0001 | <0.0001 | <0.0001 |
|  | 3-hydroxybutyrate - Liver | 0.0206 | 0.0065 | 0.7797 | 0.9901 | 0.5498 | 0.5237 | 0.2680 | 0.1701 |
| 3.A | Acetylcarnitine | <0.0001 | <0.0001 | 0.0005 | 0.0004 | 0.0026 | 0.0025 | 0.0001 | <0.0001 |
| 3.B | Carnitine | 0.0253 | 0.0241 | 0.0005 | 0.0003 | 0.0001 | <0.0001 | <0.0001 | <0.0001 |
| 3.C | Octanoylcarnitine | 0.1839 | 0.7005 | <0.0001 | <0.0001 | <0.0001 | <0.0001 | <0.0001 | <0.0001 |
| 3.D | Decenoylcarnitine | 0.2041 | 0.7776 | 0.0041 | 0.0116 | 0.0002 | 0.0002 | 0.0002 | 0.0003 |
| 3.E | Monoolein | 0.1469 | 0.5596 | 0.0002 | 0.0003 | <0.0001 | <0.0001 | 0.0004 | 0.0011 |
| 3.F | LPE 18:1 | 0.0530 | 0.2019 | 0.0015 | 0.0042 | <0.0001 | <0.0001 | 0.0001 | 0.0002 |
| 3.G | LPE 18:2 | 0.0412 | 0.1570 | <0.0001 | <0.0001 | <0.0001 | <0.0001 | <0.0001 | <0.0001 |
| 3.H | LPE 18:3 | 0.0005 | 0.0005 | <0.0001 | <0.0001 | <0.0001 | <0.0001 | <0.0001 | <0.0001 |
| 3.I | LPE 20:4 | 0.0640 | 0.2439 | <0.0001 | <0.0001 | <0.0001 | <0.0001 | 0.0012 | 0.0033 |
| 3.J | HODE | 0.0276 | 0.0263 | <0.0001 | <0.0001 | <0.0001 | <0.0001 | <0.0001 | <0.0001 |
| 3.K | DiHODE 18:2 | <0.0001 | <0.0001 | <0.0001 | <0.0001 | <0.0001 | <0.0001 | <0.0001 | <0.0001 |
|  | DiHode 18:3 | <0.0001 | <0.0001 | <0.0001 | <0.0001 | <0.0001 | <0.0001 | <0.0001 | <0.0001 |
| 3.L | DiHOME | 0.0107 | 0.0076 | <0.0001 | <0.0001 | 0.0020 | 0.0010 | 0.0225 | 0.0215 |

Statistical significance was determined using one-way ANOVA, and p-values were adjusted for multiple testing using the false discovery rate (FDR) method. Values shown are FDR-adjusted *q*-values and corresponding *p*-values. Metabolites are grouped according to the figure panels in the main text.
